# Training and assessment of non-technical skills in Norwegian helicopter emergency services: a cross-sectional and longitudinal study

**DOI:** 10.1186/s13049-018-0583-1

**Published:** 2019-01-07

**Authors:** Kristen Rasmussen, Henrik Langdalen, Stephen J. M. Sollid, Eirik Bjorheim Abrahamsen, Leif Inge K. Sørskår, Gunnar Tschudi Bondevik, Håkon B. Abrahamsen

**Affiliations:** 1 Faculty of Health Sciences, University of Stavanger, Stavanger, Norway; 2 Department of Research and Development, Norwegian Air Ambulance Foundation, Oslo, Norway; 3 Division of Critical Care, Møre and Romsdal Hospital Trust, Ålesund, Norway; 4 Department of Safety, Economics and Planning, University of Stavanger, Stavanger, Norway; 5 Division of Prehospital Medicine, Stavanger University Hospital, Stavanger, Norway; 6 Department of Global Public Health and Primary Care, University of Bergen, Bergen, Norway; 7 National Centre for Emergency Primary Health Care, Uni Research Health, Bergen, Norway; 8 Department of Anaesthesiology and Intensive Care, Stavanger University Hospital, Stavanger, Norway

**Keywords:** Air ambulances, Helicopter, Communication, Leadership, Non-technical skills, Simulation-based training

## Abstract

**Background:**

Deficient non-technical skills (NTS) among providers of critical care in helicopter emergency medical services (HEMS) is a threat to patient and operational safety. Skills can be improved through simulation-based training and assessment. A previous study indicated that physicians underwent less frequent training compared to pilots and HEMS crew members (HCM) and that all professional groups in Norwegian HEMS received limited training in how to cope with fatigue. Since then, training initiatives and a fatigue risk management project has been initiated. Our study aimed to explore if the frequency of simulation-based training and assessment of NTS in Norwegian HEMS has changed since 2011 following these measures.

**Methods:**

A cross-sectional web-based survey from October through December 2016, of physicians, HCM and pilots from all civilian Norwegian HEMS-bases reporting the overall extent of simulation-based training and assessment of NTS.

**Results:**

Of 214 invited, 109 responses were eligible for analysis. The frequency of simulation-based training and assessment of NTS has increased significantly for all professional groups in Norwegian HEMS, most prominently for the physicians. For all groups, the frequency of assessment is generally lower than the frequency of training.

**Conclusions:**

Physicians in Norwegian HEMS seem to have adjusted to the NTS training culture of the other crew member groups. This might be a consequence of improved NTS training programs. The use of behavioural marker systems systematically in HEMS should be emphasized.

**Electronic supplementary material:**

The online version of this article (10.1186/s13049-018-0583-1) contains supplementary material, which is available to authorized users.

## Introduction

Pre-hospital critical care and transport of critically ill or injured patients involve a significant risk of adverse events [1]. Studies investigating the factors contributing to critical incidents and adverse events in highly dynamic domains of healthcare, such as emergency medicine, have shown that teamwork plays an important role [2]. Team leadership is a critical skill for emergency medicine physicians directly affecting team performance and the quality of patient care [3, 4]. Poor communication has been found to be a significant factor in adverse events in air ambulance transports [5, 6], but overall, research on the causes of human errors in helicopter emergency medical services (HEMS) is still sparse [7].

Systematic training and assessment of non-technical skills (NTS) in HEMS have received little attention in the past [8, 9]. NTS can be defined as the cognitive and interpersonal skills needed to deliver safe care [10]. Seven generic categories of NTS have been suggested: situation awareness, decision-making, communication, teamwork, leadership, managing stress and coping with fatigue [11].

To document the level of simulation-based training and assessment of non-technical skills in 2011 among crew members of the Norwegian HEMS, Abrahamsen and co-workers performed a cross-sectional survey [8]. The main findings from this study was a lack of simulation-based training and assessment for all professional groups in Norwegian HEMS, that physicians underwent significantly less frequent training and assessment compared to pilots and HEMS Crew Members (HCM), and that all groups received limited training in how to cope with fatigue even though they were on call for extended hours. Since then, the Norwegian Air Ambulance Foundation has implemented a crew training camp concept for the Norwegian HEMS [12], initiated a research project of in situ simulation training during on-call hours with the implementation of weekly simulation training at several HEMS bases in Norway [13], and conducted a fatigue risk management project in Norwegian HEMS.

Our study aimed to explore if the frequency of simulation-based training and assessment of non-technical skills in Norwegian HEMS has changed following the training initiatives mentioned above. Our hypothesis is that the frequency of simulation-based training and assessment of NTS has increased in all the three professional groups.

## Methods

### Setting

Since the previous survey, one additional HEMS base has been established in Norway. The 12 HEMS bases all have helicopters staffed with a pilot, a HEMS crew member (HCM) and a physician running 24/7 services. One HEMS base is staffed with an additional flight nurse, but because the number of nurses is low, full anonymity could not be guaranteed and this professional group was not included in the previous study. This also applies to the current survey. All Norwegian HEMS physicians are certified or soon-to-be certified anaesthesiologists and employed by the local health enterprise. HCMs and pilots are employed by one of the two flight operators, Norsk Luftambulanse AS and Lufttransport RW AS.

### Questionnaire

Eight question categories regarding education and training in NTS were attached to a patient safety climate questionnaire (Additional file 1). Except for a minor adaptation in wording to also fit ground ambulance organization, the questionnaire was identical to the previous survey [8]. Similarly, our study focused on the two question categories reporting the overall extent of simulation-based training (question category I6) and assessment (question category I7) in the previous year on a four-point ordinal scale (0, 1–2, 3–5, > 5 times per year) for each of the seven generic NTS categories. The questionnaire also contained seven background variables relating to the respondents’ work characteristics; work area, geographic location, field of competence, patient contact, work hours, experience in the prehospital area and seniority in position.

### Data collection

All physicians, HCMs and pilots working in the civilian Norwegian HEMS were invited to participate in an anonymous, cross-sectional web-based survey (SurveyXact™, Rambøll Management Consulting, Oslo, Norway). A link to the survey was distributed via e-mail and five reminders were sent non-responders. The survey was open from October through December 2016.

### Statistical analysis

All answers related to simulation-based training and assessment were dichotomized into “some training/assessment” and “no training/assessment”. To visualize the development in training and assessment, ratios of the percentages from 2015 divided by the corresponding percentages from 2011, were calculated and are presented in bar charts across an ordinal scale. A ratio greater than 1, indicates a positive development in the frequency of training and assessment. To support the visuals, a series of two-sided Fisher’s exact test of the dichotomized items were performed. A *p*-value less than 0.05 should imply a rejection of the null hypothesis, which was no association between the two groups of interest and level of training and assessment. The freeware R 3.1.3 was used for all calculations and visualization producing the results presented in this paper.

### Ethical considerations

The study was approved by the Norwegian Centre for Research Data (Ref. no. 2016/45723) and was exempted from ethical approval by the Regional Committee for Medical and Health Research Western Norway (Ref. no. 2015/2249). The participants received information regarding the purpose of the study and that the questionnaires were to be treated in confidence, and their written consent to participate in the study was given at the start of the survey.

## Results

**Fig. 1:**
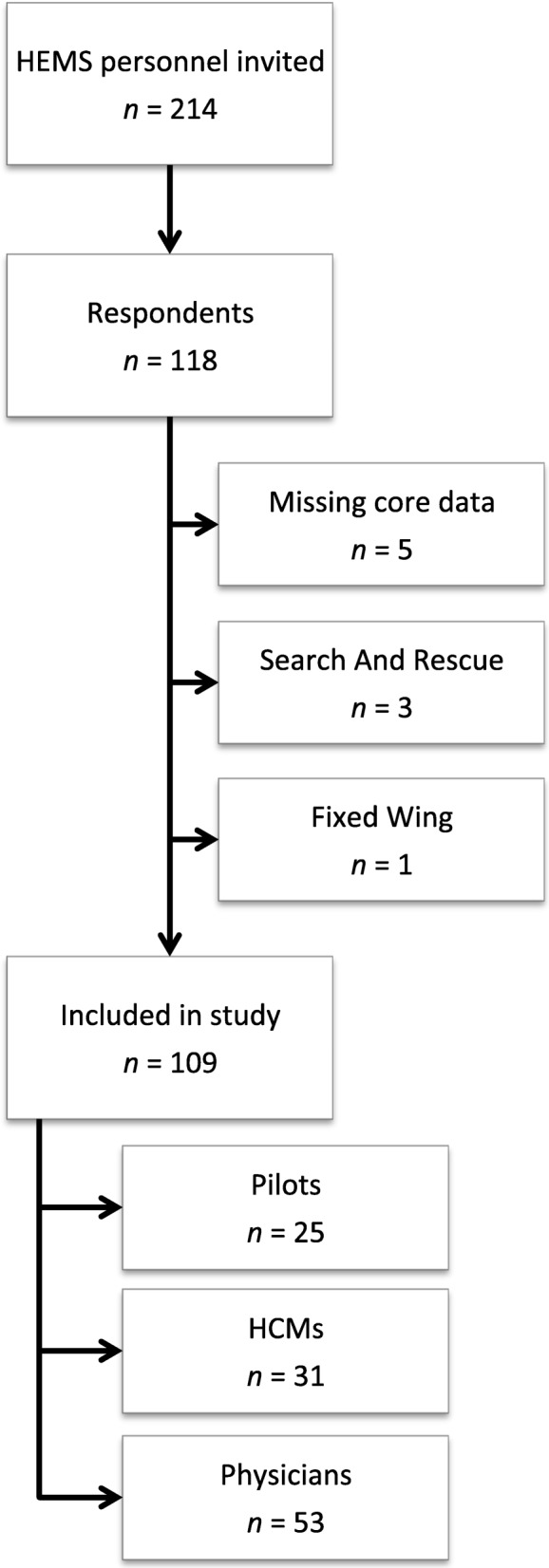
Inclusion flow chart

In total, 214 physicians, HCMs and pilots in the Norwegian civilian HEMS were invited to participate in the survey. We received 118 responses, yielding a response rate of 55.1%. Nine responses were excluded due to either missing core data, or because respondents stated search and rescue services (SAR) or fixed wing air ambulance as their main job, giving 109 responses eligible for analysis. Of these, 49% (53) were from physicians, 28% (31) from HCM and 23% (25) from pilots. In 2011, the corresponding distribution among the professional groups was 53, 27 and 20%, respectively (Table 1, Fig. 1).

**Table 1:** Demographic and professional characteristics of the study populations in 2011 and 2015

	2011 (*n* = 155)	2015 (*n* = 109)
	%	%
Professional group		
Physician	53	49
Pilot	20	23
HCM	27	28
Regional health trust		
North	14	18
Mid-Norway	22	21
West	26	21
South-East	36	39
Other	3	< 1
Prehospital experience		
Less than 1 year	5	4
1 to 5 years	19	20
6 to 10 years	27	24
11 to 15 years	16	17
16 to 20 years	15	25
21 years or more	19	10

### Overall training and assessment of NTS in Norwegian HEMS

When evaluating the results for all personnel in Norwegian HEMS as a whole, the frequency of both simulation-based training and assessment for all NTS categories have increased from 2011 to 2015. By statistical testing, we found that all changes were significant except for simulation-based training in “coping with fatigue” (Table 2).

**Table 2:** Norwegian HEMS personnel with simulation-based training in and assessment of non-technical skills

Question category	NTS category	2015 (*n* = 109)	2011 (*n* = 155)	*P*-value	
Simulation-based training of NTS	1. Decision-making	90/109 (82.6%)	87/149 (58.4%)	< 0.001	*
	2. Leadership	29/109 (73.4%)	84/150 (56.0%)	0.004	*
	3. Communication	21/109 (80.7%)	90/150 (60.0%)	< 0.001	*
	4. Situation awareness	22/109 (79.8%)	86/150 (57.3%)	< 0.001	*
	5. Teamwork	16/109 (85.3%)	99/149 (66.4%)	< 0.001	*
	6. Managing stress	32/109 (70.6%)	71/151 (47.0%)	< 0.001	*
	7. Coping with fatigue	61/109 (44.0%)	50/146 (34.2%)	0.120	
Assessment of NTS	1. Decision-making	78/109 (71.6%)	76/149 (51.0%)	0.001	*
	2. Leadership	74/109 (67.9%)	71/149 (47.7%)	0.001	*
	3. Communication	76/109 (69.7%)	69/148 (46.6%)	< 0.001	*
	4. Situation awareness	74/109 (67.9%)	69/148 (46.6%)	< 0.001	*
	5. Teamwork	81/109 (74.3%)	79/149 (53.0%)	< 0.001	*
	6. Managing stress	66/109 (60.6%)	64/149 (43.0%)	0.006	*
	7. Coping with fatigue	46/109 (42.2%)	44/146 (30.1%)	0.048	*

Number and proportion (%) of Norwegian HEMS personnel having undergone simulation-based training (question category I6) and assessment (question category I7) of seven (1–7) generic non-technical skills (NTS) in 2011 and 2015. *P-values less than 0.05 from the two-sided Fisher exact test comparing the proportions in 2011 and 2015

### Training and assessment for each professional group

**Fig. 2:**
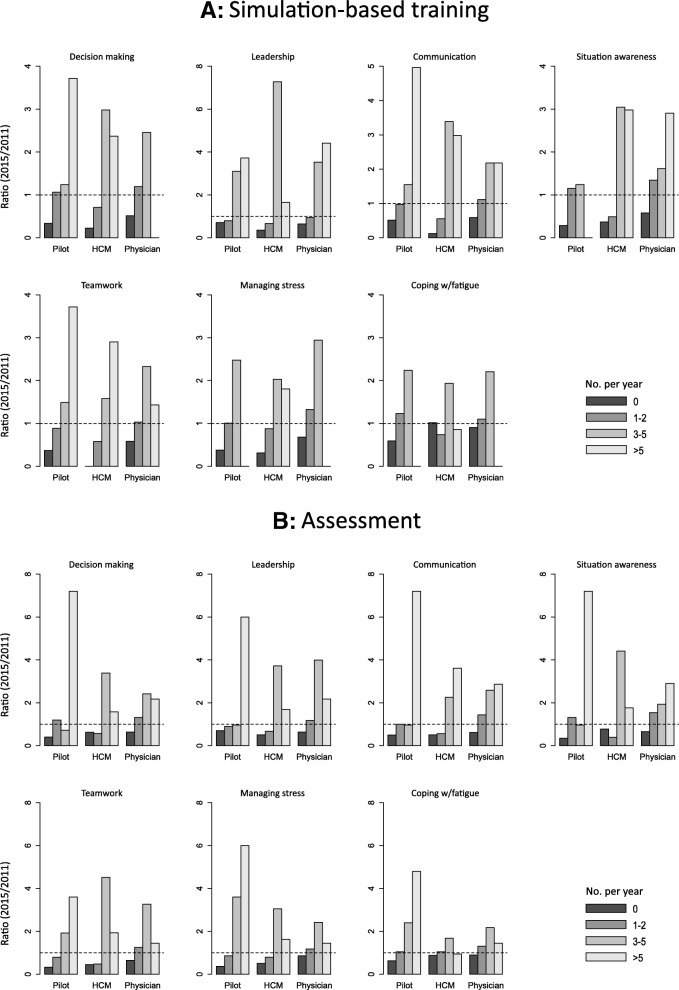
The changes in (**a**) simulation-based training in and (**b**) assessment of the generic non-technical skills within each professional group from 2011 to 2015. The ratios represent the relative frequencies (%) of 2015 divided by the relative frequencies (%) of 2011 across all four ordinal categories, with a ratio = 1 (dashed line) indicating no change in relative frequency and a ratio < 1 or > 1 respectively a decrease or an increase in frequency. Missing bars are due to categories with no data in one or both of the years surveyed, and thus, no computable ratio

Physicians were the professional group with most categories with significant increase in training and assessment from 2011 to 2015. The frequency of simulation-based training of decision-making, leadership, communication, situation awareness and managing stress has increased significantly, and physicians have been assessed significantly more frequently for all NTS except managing stress and coping with fatigue (Table 3, Fig. 2).

**Table 3:** Physicians with simulation-based training in and assessment of non-technical skills

Question category	NTS category	2015 (*n* = 53)	2011 (*n* = 82)	*P*-value	
Simulation-based training of NTS	1. Decision-making	39/53 (73.6%)	37/76 (48.7%)	0.006	*
	2. Leadership	35/53 (66.0%)	37/78 (47.4%)	0.049	*
	3. Communication	38/53 (71.7%)	40/77 (51.9%)	0.029	*
	4. Situation awareness	37/53 (69.8%)	37/77 (48.1%)	0.019	*
	5. Teamwork	40/53 (75.5%)	44/76 (57.9%)	0.060	
	6. Managing stress	28/53 (52.8%)	24/78 (30.8%)	0.018	*
	7. Coping with fatigue	16/53 (30.2%)	18/78 (23.1%)	0.419	
Assessment of NTS	1. Decision-making	32/53 (60.4%)	29/77 (37.7%)	0.013	*
	2. Leadership	31/53 (58.5%)	27/77 (35.1%)	0.012	*
	3. Communication	31/53 (58.5%)	25/76 (32.9%)	0.007	*
	4. Situation awareness	29/53 (54.7%)	24/77 (31.2%)	0.011	*
	5. Teamwork	32/53 (60.4%)	30/77 (39.0%)	0.020	*
	6. Managing stress	20/53 (37.7%)	21/77 (27.3%)	0.250	
	7. Coping with fatigue	14/53 (26.4%)	14/77 (18.2%)	0.284	

Number and proportion (%) of physicians working in Norwegian helicopter emergency medical services (HEMS) who have undergone simulation-based training (question category I6) and assessment (question category I7) of seven (1–7) generic non-technical skills (NTS) in 2011 and 2015. *P-values less than 0.05 from the two-sided Fisher exact test comparing the proportions in 2011 and 2015

In 2011, pilots reported to be assessed more frequently than physicians, while no significant difference was found regarding simulation-based training [8]. The bar plots indicate a further increase in the frequency of training and assessments for the pilots, but these changes were not significant with the exception of training and assessment of “situation awareness” and “managing stress” (Table 4, Fig. 2).

**Table 4:** Pilots with simulation-based training in and assessment of non-technical skills

Question category	NTS category	2015 (*n* = 25)	2011 (*n* = 31)	*P*-value	
Simulation-based training of NTS	1. Decision-making	22/25 (88.0%)	20/31 (64.5%)	0.064	
	2. Leadership	17/25 (68.0%)	17/31 (54.8%)	0.412	
	3. Communication	20/25 (80.0%)	19/31 (61.3%)	0.155	
	4. Situation awareness	22/25 (88.0%)	18/31 (58.1%)	0.018	*
	5. Teamwork	22/25 (88.0%)	21/31 (67.7%)	0.112	
	6. Managing stress	21/25 (84.0%)	18/31 (58.1%)	0.045	*
	7. Coping with fatigue	16/25 (64.0%)	11/28 (39.3%)	0.101	
Assessment of NTS	1. Decision-making	21/25 (84.0%)	18/30 (60.0%)	0.075	
	2. Leadership	18/25 (72.0%)	18/30 (60.0%)	0.404	
	3. Communication	20/25 (80.0%)	18/30 (60.0%)	0.147	
	4. Situation awareness	21/25 (84.0%)	16/30 (53.3%)	0.022	*
	5. Teamwork	22/25 (88.0%)	19/30 (63.3%)	0.061	
	6. Managing stress	21/25 (84.0%)	17/30 (56.7%)	0.041	*
	7. Coping with fatigue	15/25 (60.0%)	11/30 (36.7%)	0.108	

Number and proportion (%) of pilots working in Norwegian helicopter emergency medical services (HEMS) who have undergone simulation-based training (question category I6) and assessment (question category I7) of seven (1–7) generic non-technical skills (NTS) in 2011 and 2015. *P-values less than 0.05 from the two-sided Fisher exact test comparing the proportions in 2011 and 2015

HCMs appeared to be the professional group with the highest frequency of training and assessment in 2011, although not significantly different from the pilots [8]. We found a further and significant increase in the frequency of HCMs of simulation-based training in decision-making, communication, teamwork and managing stress. No significant changes were noted for assessment of any of the NTS categories. (Table 5, Fig. 2).

**Table 5:** HEMS crew members (HCM) with simulation-based training in and assessment of non-technical skills

Question category	NTS category	HCM 2015 (*n* = 31)	HCM 2011 (*n* = 42)	*P*-value	
Simulation-based training of NTS	1. Decision-making	29/31 (93.5%)	30/42 (71.4%)	0.033	*
	2. Leadership	28/31 (90.3%)	30/41 (73.2%)	0.080	
	3. Communication	30/31 (96.8%)	31/42 (73.8%)	0.010	*
	4. Situation awareness	28/31 (90.3%)	31/42 (73.8%)	0.131	
	5. Teamwork	31/31 (100.0%)	34/42 (81.0%)	0.018	*
	6. Managing stress	28/31 (90.3%)	19/42 (69.0%)	0.044	*
	7. Coping with fatigue	16/31 (51.6%)	21/40 (52.5%)	1.000	
Assessment of NTS	1. Decision-making	25/31 (80,6%)	29/42 (69.0%)	0.295	
	2. Leadership	25/31 (80,6%)	26/42 (61.9%)	0.122	
	3. Communication	25/31 (80,6%)	26/42 (61.9%)	0.122	
	4. Situation awareness	24/31 (77,4%)	29/42 (69.0%)	0.596	
	5. Teamwork	27/31 (87,1%)	30/42 (71.4%)	0.154	
	6. Managing stress	25/31 (80,6%)	26/42 (61.9%)	0.122	
	7. Coping with fatigue	17/31 (54,8%)	19/39 (48.7%)	0.638	

Number and proportion (%) of HEMS crew members (HCM) working in Norwegian helicopter emergency medical services (HEMS) who have undergone simulation-based training (question category I6) and assessment (question category I7) of seven (1–7) generic non-technical skills (NTS) in 2011 and 2015. *P-values less than 0.05 from the two-sided Fisher exact test comparing the proportions in 2011 and 2015

### Training and assessment based on employer

The crew members can be separated with respect to employer. Of the respondents, 49% were employed by the flight operator (HCMs and pilots) and 51% were working for the health enterprise (physicians) compared to 47 and 53%, respectively in the previous survey [8].

In 2011, health enterprise employees experienced significantly less frequent training and assessment than flight operator personnel for all NTS categories [8]. In our study, flight operator employees were reporting a significant increase in the frequency of both training and assessment of all NTS except “leadership” and “coping with fatigue” (Table 6). Even though the physicians were the group with most categories with significant increase in training and assessment in the period (Table 3), the significant differences based on employment status still exist for all categories except “leadership” (Table 6).

**Table 6:** Flight operator employees and health enterprise employees with simulation-based training in and assessment of non-technical skills

Question category	NTS category	Flight 2015	Flight 2011	*P*-value		Health 2015	*P*-value	
		(*n* = 56)	(*n* = 73)	A		(*n* = 53)	B	
Simulation-based training of NTS	1. Decision-making	51/56 (91.1%)	50/73 (68.5%)	0.002	*	39/53 (73.6%)	0.022	*
	2. Leadership	45/56 (80.4%)	47/72 (65.3%)	0.075		35/53 (66.0%)	0.129	
	3. Communication	50/56 (89.3%)	50/73 (68.5%)	0.006	*	38/53 (71.7%)	0.028	*
	4. Situation awareness	50/56 (89.3%)	49/73 (67.1%)	0.003	*	37/53 (69.8%)	0.016	*
	5. Teamwork	53/56 (94.6%)	55/73 (75.3%)	0.003	*	40/53 (75.5%)	0.006	*
	6. Managing stress	49/56 (87.5%)	47/73 (64.4%)	0.004	*	28/53 (52.8%)	< 0.001	*
	7. Coping with fatigue	32/56 (57.1%)	32/68 (47.1%)	0.284		16/53 (30.2%)	0.007	*
Assessment of NTS	1. Decision-making	46/56 (82.1%)	47/72 (65.3%)	0.045	*	32/53 (60.4%)	0.019	*
	2. Leadership	43/56 (76.8%)	44/72 (61.1%)	0.085		31/53 (58.5%)	0.064	
	3. Communication	45/56 (80.4%)	44/72 (61.1%)	0.021	*	31/53 (58.5%)	0.021	*
	4. Situation awareness	45/56 (80.4%)	45/71 (63.4%)	0.049	*	29/53 (54.7%)	0.007	*
	5. Teamwork	49/56 (87.5%)	49/72 (68.1%)	0.012	*	32/53 (60.4%)	0.002	*
	6. Managing stress	46/56 (82.1%)	43/72 (59.7%)	0.007	*	20/53 (37.7%)	< 0.001	*
	7. Coping with fatigue	32/56 (57.1%)	30/69 (43.5%)	0.152		14/53 (26.4%)	0.002	*

Number and proportion (%) of Norwegian HEMS personnel employed by the flight operator and health enterprise who have undergone simulation-based training (question category I6) and assessment (question category I7) of seven (1–7) generic non-technical skills (NTS).*P-values less than 0.05 from the two-sided Fisher exact test comparing (A) the proportions of flight operator employees in 2011 and 20 and (B) flight operator employees with health enterprise employees in 2015

## Discussion

### Training of non-technical skills

To deliver high quality of care and patient safety, training in technical skills is important to be competent in critical care procedures [14]. Non-technical skills are essential to complement the technical skills in a work setting such as HEMS. Deficiencies in communication and teamwork are frequent contributors to adverse events in health care [15]. There is also increasing awareness about the positive influence of teamwork on clinical performance [16, 17] and clinical outcomes [18, 19].

Even though the theoretical basis and the evidence regarding educational methods to enhance patient safety using NTS training are still limited [10], both simulation and classroom-based training has been found to improve teamwork processes [15]. An interdisciplinary team training program using in-situ simulation gave a statistically significant and persistent improvement in perinatal morbidity [20]. Similar results have been found in surgical outcome after team training of operating room personnel [19]. Simulation-based team training seems to be the most prominent mode of training in the literature [15].

Duration and frequency of training varies, and there is currently limited, but emerging, evidence that provides insight into the frequency of retraining needed to maintain effective teamwork skills [15]. Significant improvement has been found for critical care providers at 6 and 12 months post-training [21], and studies on simulation based training in neonatal resuscitation seems to favour low dose, high frequency training [22]. This points in the direction of at least annual training, similar to common practice for crew resource management (CRM) training in aviation.

The content and schedule of training in technical skills need to be tailored due to variations in mission profiles and exposure to different procedures [14]. Human errors, on the other hand, are not limited to inexperienced clinicians, and NTS training is therefore equally important to all. So far, a consensus regarding the content of team training has not been achieved, but the most commonly targeted teamwork competencies are communication, situational awareness and leadership [15]. In addition to these, decision-making, teamwork, managing stress and coping with fatigue are often included in non-technical skills evaluation schemes.

### Assessment versus training

Assessment is the process of observing, recording, interpreting and evaluating individual performance and serves different purposes: to audit the level of skills of individuals or units, but also to evaluate training programs [11]. A number of non-technical skills rating frameworks, behavioural marker systems, have been developed for health-care domains closely related to the air ambulance setting [23–26], but a tool for assessment of non-technical skills for HEMS such as the AeroNOTS, has just recently been developed and yet not fully validated [27]. Generally, the frequency of assessment was lower than the frequency of simulation-based training for all three professional groups in our study. This result underlines the undone work in using assessment tools systematically in HEMS.

### Training in Norwegian HEMS

Norwegian HEMS providers have a contractual mandatory training program in rescue and flight operative procedures, including recurrent flight simulator training for pilots and HCMs. Medical training, simulation-based or otherwise, depend on local initiative and commitment. In the study of Abrahamsen, physicians underwent significantly less frequent simulation-based training compared to the other groups [8]. In our study, physicians were the one group with a significant increase in most NTS categories, and thus, an important contributor to the overall increase in the frequency of training in the Norwegian HEMS. The before-mentioned initiatives with in-situ simulation [13] and the all crew training camp [12] may be one explanation to this result. The proportion of physicians training currently seems to be at the level of the other groups in 2011, but they still train significantly less than flight operative employees. Thus, a great potential for simulation-based training still exists among the HEMS physicians.

### Coping with fatigue

The results from the different professional groups were inconsistent regarding each of the generic NTS, and with the limitation in response rate and sample size in our survey, these results should not be over-interpreted. For *coping with fatigue*, on the other hand, we did not find significant increase for any professional group, despite the finding from 2011 where all professional groups received limited training. This may be seen as a paradox since the non-technical performance of critical care air transfer clinicians is impaired when they are fatigued [28], and fatigue training seems to improve safety and health outcome for EMS personnel [29]. Fatigue and stress management are usually included in training programs, although it has been questioned whether it is appropriate to include these topics in assessment schemes of NTS. Both can be difficult to detect and rate unless extreme symptoms are displayed, in which other skills will be affected [11]. Another influencing factor may be the lack of a consensus on the definition of fatigue and a standardized survey instrument to measure fatigue among EMS worker groups. Only a limited number of tools used in other settings for assessment of fatigue exist, and research focused on development and testing of fatigue survey instruments tailored specifically for emergency medical services is needed [30]. The on-going research project in Norwegian HEMS in fatigue risk management will hopefully contribute to developing useful tools for fatigue training and assessment.

### Limitations

Our study was part of a combined survey of both ground and air ambulance with more than 5000 invited participants, and thus, the same follow up with personal reminders to all invited as the survey of Abrahamsen [8], was not feasible. Our response rate is therefore noticeably lower, but the distribution in professional groups, prehospital experience and geographical location was largely similar (Table 1). We do not know, however, if personnel who have undergone training were more likely to respond to our survey or not, which could result in a non-responder bias and possibly more significant changes than otherwise. The results should be interpreted according to these limitations with an emphasis on the major lines and not detailed results.

In both surveys, respondents were asked to report exclusively on the frequency of interdisciplinary prehospital simulation training. We cannot, nevertheless, exclude that pilots and HCMs may have reported on mandatory flight operative training and that this may explain the better results for these groups in both surveys. We also cannot exclude that physicians may have reported on intra-hospital training.

When asked retrospective to specify the number of training sessions and assessments, some uncertainty must be expected. We have mainly based our conclusions on the dichotomized data, “no training” or “some training”, which we have assumed more reliable. Ideally, a longer period between the two surveys would be preferable. This was not possible as our study was a part of a larger research project.

Finally, as discussed earlier, in order to fully understand the effect of simulation training on patient outcome, further research is needed.

## Conclusion

The frequency of simulation-based training and assessment of NTS has increased significantly in Norwegian HEMS. Physicians seem to be adjusting to the training culture of other professional groups in HEMS, but still, there is a great potential for improving training frequency and volume among the HEMS physicians. Systematic assessment of NTS, including fatigue management, should be a future focus area in HEMS.

## Additional file

Additional file 1: Questionnaire (English translation). (PDF 180 kb)

## Abbreviations

AeroNOTS: Aeromedical non-technical skills
HCM: HEMS Crew Member
HEMS: Helicopter Emergency Medical Service
NTS: Non-technical skills
SAR: Search and rescue
