# Supplementary material for: Training and assessment of non-technical skills in Norwegian helicopter emergency services: a cross-sectional and longitudinal study
Source: Scand J Trauma Resusc Emerg Med. 2019 Jan 7;27:1. doi: 10.1186/s13049-018-0583-1 (PMC6323750; doi:10.1186/s13049-018-0583-1)
Supplement: Supplementary file 1 — Questionnaire (English translation). (PDF 180 kb) [file 13049_2018_583_MOESM1_ESM.pdf]

Thank you for taking the time to respond to this **national survey** from the University of Stavanger.

The survey maps **your opinion** on patient safety, adverse events and incident reporting in your prehospital service. Your response will be treated strictly confidentially and your identity will not be traceable. The questionnaire should take approximately 15 minutes to complete.

Read the statements carefully. Be honest when answering. For each of the statements choose the one that fits best.

With regards
Leif Inge K. Sørskår
University of Stavanger

I have received information about the study and I am willing to participate

- ☐ Yes
- ☐ No

J: Background information

What is your primary work area? Select ONE Answer or specify. (If more primary work areas, choose the option that best fits what you want to answer.)

- ☐ Ground EMS/ambulance
- ☐ Physician manned rapid response car/ambulance
- ☐ Ambulance boat
- ☐ Helicopter emergency medical service (HEMS)
- ☐ Search and rescue helicopter (SAR)
- ☐ Fixed wing air ambulance
- ☐ Other, please specify: _____

A: Your work area/unit and patient safety

Definitions:

- ***"Your local unit"*** *is defined as the unit where you primarily work. EXAMPLE: An ambulance station, a pre-hospital base or department or similar located in the same geographical location.*
- *An **"adverse event"** is defined as an accidental event due to medical examination and/or treatment.*

Please indicate your agreement or disagreement with the following statements regarding your own local unit.

	Strongly disagree	Disagree	Neither	Agree	Strongly agree
People support one another in our local unit	☐	☐	☐	☐	☐
We have enough staff to handle the workload	☐	☐	☐	☐	☐
When a lot of work needs to be done quickly, we work together as a team to get the work done	☐	☐	☐	☐	☐
In our local unit, people treat each other with respect	☐	☐	☐	☐	☐
Staff in our local unit work longer hours than is best for patient care	☐	☐	☐	☐	☐
We are actively doing things to improve patient safety	☐	☐	☐	☐	☐
We use more agency/temporary staff than is best for patient care	☐	☐	☐	☐	☐
Staff feel like their mistakes are held against them	☐	☐	☐	☐	☐
Mistakes have led to positive changes here	☐	☐	☐	☐	☐
It is just by chance that more serious mistakes do not happen in our local unit.	☐	☐	☐	☐	☐
When one area in this unit gets really busy, others help out	☐	☐	☐	☐	☐
When an event is reported, it feels like the person is being written up, not the problem	☐	☐	☐	☐	☐
After we make changes to improve patient safety, we evaluate their effectiveness	☐	☐	☐	☐	☐

We work in "crisis mode" trying to do too much, too quickly	☐	☐	☐	☐	☐
Patient safety is never sacrificed to get more work done	☐	☐	☐	☐	☐
Staff worry that mistakes they make are kept in their personnel file	☐	☐	☐	☐	☐
We have patient safety problems in our local unit	☐	☐	☐	☐	☐
Our procedures and systems are good at preventing errors from happening	☐	☐	☐	☐	☐
I will ask my colleagues to stop work I consider is done in an unsafe manner	☐	☐	☐	☐	☐
I will report if I become aware of a dangerous situation	☐	☐	☐	☐	☐

B: Safety of employees

Please indicate your agreement or disagreement with the following statements regarding your own local unit.

	Strongly disagree	Disagree	Neither	Agree	Strongly agree
My colleagues will stop me if I work in an unsafe manner	☐	☐	☐	☐	☐
I will stop doing my job if I think it might be dangerous for me or others to continue	☐	☐	☐	☐	☐

C: Your supervisor/manager

Definition:
*The terms **"with us"** and **"management"** refer to the local unit where you primarily work, and to the management in this unit, respectively.*

Please indicate your agreement or disagreement with the following statements about your immediate supervisor/manager or person to whom you directly report in your local unit.

	Strongly disagree	Disagree	Neither	Agree	Strongly agree
My local supervisor/manager says a good word when he/she sees a job done according to established patient safety procedures	☐	☐	☐	☐	☐
My local supervisor/manager considers staff suggestions for improving patient safety	☐	☐	☐	☐	☐
Whenever pressure builds up, my local supervisor/manager wants us to work faster, even if it means taking shortcuts	☐	☐	☐	☐	☐
My local supervisor/manager ignores patient-safety problems that happen over and over	☐	☐	☐	☐	☐

D: Communication

How often do the following things happen in your work area/local unit?

	Strongly disagree	Disagree	Neither	Agree	Strongly agree
We are given feedback about changes put into place based on event reports	☐	☐	☐	☐	☐
Staff will freely speak up if they see something that may negatively affect patient care	☐	☐	☐	☐	☐
We are informed about errors that happen in this unit	☐	☐	☐	☐	☐
Staff feel free to question the decisions or actions of those with more authority	☐	☐	☐	☐	☐
In my local unit we discuss ways to prevent errors from happening again	☐	☐	☐	☐	☐
Staff are afraid to ask questions when something does not seem right	☐	☐	☐	☐	☐

E: Your evaluation of the patient safety

Please give your work area/local unit an overall grade on patient safety.

- ☐ Excellent
- ☐ Very Good
- ☐ Acceptable
- ☐ Poor
- ☐ Very poor

F: Frequency of events reported

In your work area/local unit, when the following incidents happen, how often are they reported?

	Never	Rarely	Sometimes	Most of the time	Always
When a mistake is made, but is caught and corrected before affecting the patient, how often is this reported?	☐	☐	☐	☐	☐
When a mistake is made, but has no potential to harm the patient, how often is it reported?	☐	☐	☐	☐	☐
When a mistake is made that could harm the patient, but does not, how often is this reported?	☐	☐	☐	☐	☐

G: Number of events reported

In the past 12 months, how many event reports have you filled out and submitted?

- ☐ No rapports
- ☐ 1-2 rapports
- ☐ 3-5 rapports
- ☐ 6-10 rapports
- ☐ 11-20 rapports
- ☐ 21 rapports or more

H: The pre-hospital system

Please indicate your agreement or disagreement with the following statements about your pre-hospital system. (Ground EMS, HEMS dispatch center, emergency department, GP on call etc.)

	Strongly disagree	Disagree	Neither	Agree	Strongly agree
Hospital management provides a work climate that promotes patient safety	☐	☐	☐	☐	☐
Pre-hospital units do not coordinate well with each other	☐	☐	☐	☐	☐
Things "fall between the cracks" when transferring patients from one unit to another	☐	☐	☐	☐	☐
There is good cooperation among units that need to work together	☐	☐	☐	☐	☐
Important patient care information is often lost during patient handover	☐	☐	☐	☐	☐
It is often difficult to work with staff from other units in the prehospital system	☐	☐	☐	☐	☐
Problems often occur in the exchange of information across prehospital units	☐	☐	☐	☐	☐
The actions of hospital management show that patient safety is a top priority	☐	☐	☐	☐	☐
Hospital management seems interested in patient safety only after an adverse event happens	☐	☐	☐	☐	☐
Prehospital units work well together to provide the best care for patients	☐	☐	☐	☐	☐
Handovers are problematic for patients in this prehospital system	☐	☐	☐	☐	☐

I: Education and training

Do you feel that your pre-hospital skills are deficient related to challenges you have to face in your prehospital work?

	Deficient	NOT deficient
Decision-making	☐	☐
Leadership	☐	☐
Communication	☐	☐
Situation awareness	☐	☐
Teamwork	☐	☐
Managing stress	☐	☐
Coping with fatigue	☐	☐

How many times during 2015 did you participate in multidisciplinary pre-hospital simulation-based training of one or more of the skills below, along with your professional partners?

	0 times	1-2 times	3-5 times	More than 5 times
Decision-making	☐	☐	☐	☐
Leadership	☐	☐	☐	☐
Communication	☐	☐	☐	☐
Situation awareness	☐	☐	☐	☐
Teamwork	☐	☐	☐	☐
Managing stress	☐	☐	☐	☐
Coping with fatigue	☐	☐	☐	☐

How many times during 2015 were the following of your pre-hospital skills systematically observed and evaluated?

	0 times	1-2 times	3-5 times	More than 5 times
Decision-making	☐	☐	☐	☐
Leadership	☐	☐	☐	☐
Communication	☐	☐	☐	☐
Situation awareness	☐	☐	☐	☐
Teamwork	☐	☐	☐	☐
Managing stress	☐	☐	☐	☐
Coping with fatigue	☐	☐	☐	☐

Where is your primary prehospital unit located? Select ONE option or please specify.

☐ Nordlandssykehuset HF
☐ Universitetssykehuset Nord-Norge HF
☐ Helgelandssykehuset HF
☐ Finnmarkssykehuset HF
☐ St. Olavs Hospital HF
☐ Helse Nord-Trøndelag HF
☐ Helse Møre og Romsdal HF
☐ Helse Fonna HF
☐ Helse Førde HF
☐ Helse Stavanger HF

- ☐ Helse Bergen HF
- ☐ Oslo Universitetssykehus HF
- ☐ Vestre Viken HF
- ☐ Sørlandet sykehus HF
- ☐ Sykehuset Innlandet HF
- ☐ Sykehuset Telemark HF
- ☐ Sykehuset Vestfold HF
- ☐ Sykehuset Østfold HF
- ☐ Other, please specify: _____

What is your staff position? Select one answer that best describes your staff position.

- ☐ EMT with authorization
- ☐ Paramedic (in-house training)
- ☐ Paramedic (university college)
- ☐ HEMS Crew Member (HCM)
- ☐ Nurse anaesthetist (with EMT authorization)
- ☐ Nurse anaesthetist (w/o EMT authorization)
- ☐ Intensive care nurse (with EMT authorization)
- ☐ Intensive care nurse (w/o EMT authorization)
- ☐ Nurse (with EMT authorization)
- ☐ Nurse (w/o EMT authorization)
- ☐ Physician in training, anaesthesiology
- ☐ Physician, anaesthesiologist
- ☐ Other, please specify: _____

In your staff position, do you typically have direct interaction or contact with patients?

- ☐ YES, I typically have direct interaction or contact with patients.
- ☐ NO, I typically do NOT have direct interaction or contact with patients.

How long have you worked in the pre-hospital system?

- ☐ Less than 1 year
- ☐ 1 to 5 years
- ☐ 6 to 10 years
- ☐ 11 to 15 years
- ☐ 16 to 20 years
- ☐ 21 years or more

How many consecutive hours do your regularly scheduled on-call duty last at most?

- ☐ Up to 8 hours
- ☐ 9 - 12 hours
- ☐ 13 - 16 hours
- ☐ 17 - 24 hours
- ☐ 25 - 48 hours
- ☐ 49 - 72 hours
- ☐ 4 to 7 days
- ☐ More than 7 days – please specify: _____

How long have you worked in your current specialty or profession?

- ☐ Less than 1 year
- ☐ 1 to 5 years
- ☐ 6 to 10 years
- ☐ 11 to 15 years
- ☐ 16 to 20 years
- ☐ 21 years or more

K: Your comments

Which are the three most prevalent adverse events you have observed or caused yourself in the pre-hospital environment?

Which are the three measures that you think could improve pre-hospital patient safety?

Please feel free to write any comments about patient safety, error, or event-reporting in your pre-hospital system.

Thank you for completing the survey!
